# Retrograde regulation of STIM1-Orai1 interaction and store-operated Ca^2+^ entry by calsequestrin

**DOI:** 10.1038/srep11349

**Published:** 2015-06-18

**Authors:** Limin Wang, Lane Zhang, Shu Li, Yuanyuan Zheng, Xinxin Yan, Min Chen, Haoyang Wang, James W. Putney, Dali Luo

**Affiliations:** 1 Department of Pharmacology, Capital Medical University, Beijing 100069, P.R. China; 2 Laboratory of Signal Transduction, National Institute of Environmental Health Sciences, National Institutes of Health, Research Triangle Park, NC 27709, USA

## Abstract

Interaction between the endoplasmic reticulum (ER)-located stromal interaction molecue1 (STIM1) and the plasma membrane-located Ca^2+^ channel subunit, Orai1, underlies store-operated Ca^2+^ entry (SOCE). Calsequestrin1 (CSQ1), a sarcoplasmic reticulum Ca^2+^ buffering protein, inhibits SOCE, but the mechanism of action is unknown. We identified an interaction between CSQ1 and STIM1 in HEK293 cells. An increase in monomeric CSQ1 induced by depleted Ca^2+^ stores, or trifluoperazine (TFP), a blocker of CSQ folding and aggregation, enhanced the CSQ1-STIM1 interaction. In cells with Ca^2+^ stores depleted, TFP further increased CSQ1 monomerization and CSQ1-STIM1 interaction, but reduced the association of STIM1 with Orai1 and SOCE. Over-expression of CSQ1 or a C-terminal (amino acid 388–396) deletion mutant significantly promoted the association of CSQ1 with STIM1, but suppressed both STIM1-Orai1 interaction and SOCE, while over-expression of the C-terminal (amino acid 362–396) deletion mutant had no effect. The physical interaction between low polymeric forms of CSQ1 and STIM1 likely acts by interfering with STIM1 oligimerization and inhibits STIM1-Orai1 interaction, providing a brake to SOCE under physiological conditions. This novel regulatory mechanism for SOCE may also contribute to the pathological Ca^2+^ overload in calsequestrin deficient diseases, such as malignant hyperthermia and ventricular tachycardia.

Store-operated Ca^2+^ entry (SOCE) serves as a pivotal Ca^2+^ influx pathway in non-excitable cells. It is essential for the mediation of diverse cell functions such as cell activation, proliferation, differentiation, growth and cytokine production and release^1^,^2^. The major known molecular components of the SOCE mechanism include the endoplasmic reticulum (ER) membrane-located stromal interaction molecue1 (STIM1) and the plasma membrane-located Ca^2+^ release-activated Ca^2+^ channel pore forming subunit, Orai1^3^,^4^. Upon depletion of ER Ca^2+^ stores, STIM1 senses the lowered ER Ca^2+^content through its amino terminus in the ER lumen and then oligomerizes and redistributes to an ER membrane compartment closely juxtaposed to the adjacent plasma membrane, forming STIM1 clusters^5^,^6^. Subsequently, the carboxy-terminal cytoplasmic region of STIM1 interacts physically with Orai1 and thus enables Orai1 gating and robust Ca^2+^ influx from the extracellular space^7^,^8^,^9^.

Although the basic mechanism by which STIM1 activates Orai1 channels is fairly well understood, the cellular machinery that regulates and modulates STIM1-Orai1 interaction and SOCE is not so well known. Recent studies have revealed several modulators of the STIM1-Orai1 complex, located either in the plasma or ER lumen. For examples, STIM2, ER Ca^2+^ ATPase 2 (SERCA2), the microtubule plus-end binding protein (EB1), Ca^2+^ release activated Ca^2+^ channel regulator2A, surfeit locus protein4 and septins have been reported to interact with STIM1^10^,^11^,^12^.

Calsequestrin (CSQ) is a major Ca^2+^ buffering protein in the sarcoplasmic reticulum (SR). It has two isoforms, CSQ1 (skeletal) and CSQ2 (cardiac)^13^,^14^. Earlier studies have presumed that CSQ simply functions as a high capacity, moderate affinity Ca^2+^ buffer. However, more recent studies have demonstrated that this SR protein also plays a key role in regulating the ryanodine receptor (RyR)-mediated Ca^2+^ release in skeletal muscle cells. It acts as a luminal Ca^2+^ sensor in regulating RyR activities via its interactions with triadin and junctin^15^,^16^,^17^. CSQ molecules exist as monomers when luminal Ca^2+^ concentration is lower than 10 μM. As luminal Ca^2+^ concentration increases, it undergoes molecule compaction, dimerization and polymerization^18^,^19^. The polymer of CSQ is stable and anchored to the SR membrane at a luminal Ca^2+^ concentration of 1 mM through interaction of its carboxyl-terminal aspartate-rich region, a main Ca^2+^ binding motif, with triadin or junctin^15^,^17^,^20^,^21^. Abnormalities in *CSQ* gene expression may result in cardiac arrhythmias and malignant hyperthermia^22^,^23^. In addition to regulating Ca^2+^ release in skeletal muscle cells, CSQ1 can also function as an inhibitor of SOCE^24^,^25^. CSQ1 lacking the asp-rich terminal (amino acids 352–367) failed to inhibit SOCE, suggesting a role for a domain in the C-terminal region^25^. However, the mechanism through which CSQ1 exerts its inhibitory effects on SOCE is not known.

Unlike skeletal muscle, the function of CSQ in non-muscle cells is rather unclear. In a previous study, we reported that CSQ1 is expressed in non-muscle cells and functions as a regulator of Ca^2+^ release as in muscle^26^. Here we report that over-expression of both human wild-type CSQ1 and a C-terminal deletion mutant, C9 (deletion of wild-type amino acids 388–396), can suppress SOCE after Ca^2+^ store depletion induced by thapsigargin (TG) in HEK293 cells, while over-expression of the C-terminal deletion mutant C35 (deletion of amino acids 362–396) has no effect. Furthermore, we report, for the first time, that a direct physical interaction between CSQ1 and STIM1 prevents STIM1 interaction with Orai1 and thus down-regulates SOCE.

## Results

### CSQ1 associates with STIM1 in the ER

**Figure 1:**
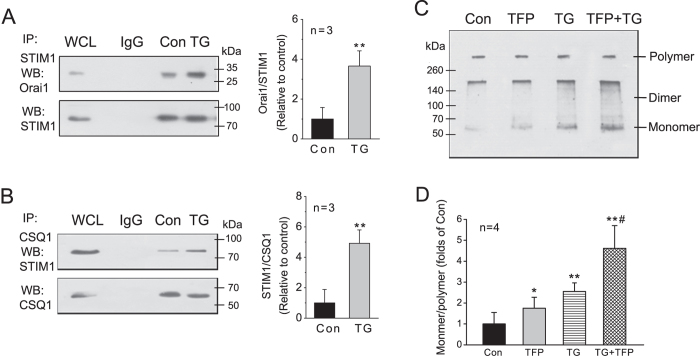
CSQ1 interacts with STIM1 in HEK293 cells. HEK293 cells cultured in FBS-containing media for 24 h were incubated in FBS-free DMEM for 5 h, and then cells were suspended in Ca^2+^-free Hepes-buffered saline solution (HBSS, 0.1 mM EGTA added) for 5 min and stimulated with or without 1 μM TG for 5 min until harvested. (**A**) Co-immunoprecipitations between STIM1 and Orai1. Whole cell lysates (WCL) were immunoprecipitated with specific anti-STIM1monoclonal mouse antibody (mAb) and protein-G agarose, then Western blot was performed with specific anti-Orai1mAb (left). Equal amounts of normal mouse IgG was used as an immunoprecipitation antibody substitute for negative control. Membranes were reprobed with the antibody used for immunoprecipitation for protein loading control in all the co-immunoprecipitation experiments. STIM1-Orail1 association intensities were quantified as average protein ratios of Orai1/STIM1 (right). (**B**) Co-immunoprecipitation between CSQ1 and STIM1. Whole cell lysates were immunoprecipitated with specific anti-CSQ1 mAb and protein-G agarose, then Western blot was performed with specific anti-STIM1mAb (left). STIM1-CSQ1 association intensities were quantified as average protein ratios ± SD of STIM1/CSQ1 (right). **p < 0.01 vs. control, n = 3 for both A and B. (**C**) Conformation of CSQ1 is influenced by TFP or TG treatment. HEK293 cells were pre-incubated with or without 20 μM TFP for 10 min, then were suspended in Ca^2+^-free HBSS for 5 min and stimulated with or without 1 μM TG for 5 min. All the cells were cross-linked with 2% formaldehyde for 5 min, lysed and subjected to 10% SDS-PAGE and subsequent Western blotting with anti-CSQ1  mAb. (**D**) The antibody-reacting bands of CSQ1 were quantified and the average ratios of monomer/polymer CSQ1 are represented as means ± SD. N = 4 for each bar, *p < 0.05 and **p < 0.01 vs. control; ^#^p < 0.05 vs. TG group.

We have investigated the relationship between CSQ1 and the essential components of SOCE in HEK293 cells, a cell line in which functional CSQ1 but not CSQ2 expression has been reported^26^ (supplemental Fig. 1). As previously shown^27^, physical interaction between STIM1 and Orai1 was markedly increased upon depletion of Ca^2+^ stores by 1 μM TG (Fig. 1A), and a robust Ca^2+^ entry through the gating of SOC channels was observed (data not shown). CSQ1 also co-immunoprecipitated with STIM1, and this association was enhanced by TG stimulation (Fig. 1B). These data indicate that both the CSQ1-STIM1 and STIM1-Orai1 interactions increase with Ca^2+^ store depletion, suggesting that CSQ1 may be affecting SOCE through its interaction with STIM1.

### Monomeric CSQ1 interacts with STIM1

It is well known that the polymeric status of CSQ is regulated by the level of SR Ca^2+^. CSQ molecules become dimerized and then polymerized as the Ca^2+^ concentration increases from 10 μM to 1 mM^19^,^20^,^21^. However, it is not clear whether CSQ remains polymerized or reverts to monomers upon ER Ca^2+^ depletion. Here, we used trifluoperazine (TFP), a CSQ inhibitor that binds to CSQ and inhibits its folding, compaction and the subsequent aggregation^21^,^28^, to assess the relationship between CSQ conformation and its association with STIM1. We conducted protein chemical cross-linking experiments [see Methods], which showed that TFP (20 μM) significantly increased the monomer proportion of CSQ1 (Fig. 1C,D). TG at a concentration of 1 μM that increased CSQ1-STIM1 association also increased the monomer proportion of CSQ1, and addition of TFP 10 min prior to TG increased the monomers even further compared with TG stimulation alone (Fig. 1C,D).

**Figure 2:**
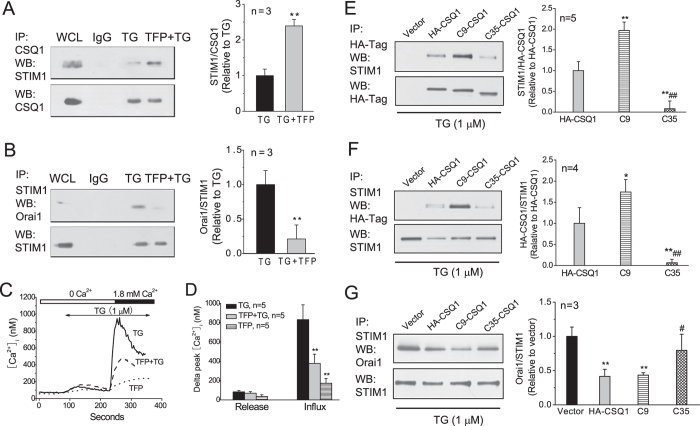
TFP enhances CSQ1-STIM1 association but inhibits STIM1-Orai1 interaction and SOCE, and deletion of carboxyl-terminal 35 amino acids in CSQ1 attenuates CSQ1 binding to STIM1. (**A**) Effect of TFP on the association of CSQ1 with STIM1. HEK293 cells were preincubated with (TG+TFP group) or without (TG group) 20 μM TFP for 10 min, then were suspended in Ca^2+^-free HBSS for 5 min and stimulated with 1 μM TG for another 5 min. Whole cell lysates were immunoprecipitated with anti-CSQ1 mAb and protein-G agarose, and then Western blot was performed with anti-STIM1 mAb (left). CSQ1-STIM1 association intensities were quantified as average protein ratios ± SD of STIM1/CSQ1 (right). (**B**) Effect of TFP on the association of STIM1 with Orai1, procedure as in A. **p < 0.01 vs. TG group, n = 3 for both **A** and **B**. (**C**) TFP impacts [Ca^2+^]_i_ response to TG. TG induced Ca^2+^ mobilization in Ca^2+^-free HBSS (0.1 mM EGTA) and Ca^2+^ entry following addition of 1.8 mM Ca^2+^ in Fura2-loaded HEK293 cells. (**D**) Averaged delta peak [Ca^2+^]_i_ (minus the basal [Ca^2+^]_i_) ± SD from experiments shown in **C**. N = 5 independent experiments for each bar, **p<0.01 vs. TG group. (**E**) Co-immunoprecipitation of exogenous CSQ1 and endogenous STIM1. HEK293 cells expressing empty vectors or exogenous HA-CSQ1, C9 or C35 truncations were suspended in Ca^2+^-free HBSS and then stimulated with 1 μM TG for 5 min. Immunoprecipitation and Western blot as indicated (left). STIM1-HA-CSQ1 association intensities were quantified as average protein ratios ± SD of STIM1/HA-Tag (right). (**F**) Reverse co-immunoprecipitation between exogenous HA-CSQ1 and STIM1. Immunoprecipitation and Western blot as indicated (left). HA-Tag-STIM1 association intensities were quantified as average protein ratios ± SD of HA-Tag/STIM1 (right). (**G**) Co-immunoprecipitation between STIM1 and Orai1. Immunoprecipitation and Western blot as indicated (left). STIM1-Orai1 association intensities were quantified as average protein ratios ± SD of Orai1/STIM1 (right). *p < 0.05 and **p < 0.01 vs. HA-CSQ1 (E and F) or vector group (G); ^#^p < 0.05 and ^##^p < 0.01 vs. C9-CSQ1 in all the panels.

Additionally, pretreatment of HEK293 cells with TFP substantially increased the co-immunoprecipitation of CSQ1 and STIM1 mediated by TG in Ca^2+^-free medium (Fig. 2A), implying that it is the monomer form of CSQ1 that interacts with STIM1. To investigate whether the CSQ1 interaction with STIM1 influenced STIM1-Orai1 interaction, co-immunoprecipitation between STIM1 and Orai1 was examined and revealed that TFP lowered the STIM1-Orai1 association induced by TG and accordingly reduced TG-induced SOCE (Fig. 2B–D). TFP (20 μM) also caused observable increases in [Ca^2+^]_i_ in Ca^2+^-free medium and upon restoring the extracellular Ca^2+^ presumably because of its inhibition of Ca^2+^ binding with CSQ. Pretreatment of cells with 20 μM TFP for 10 min significantly suppressed the TG-induced Ca^2+^ influx but not the Ca^2+^ release phase. Immunohistochemical localization of endogenous STIM1 and CSQ1 also revealed co-localization of STIM1 and CSQ1 by TG, and further increase in co-localization by TFP (Supplemental Fig. 2).

Since TFP also inhibits calmodulin at high concentrations^29^, we compared the effect of TFP with that of N-(6-aminohexyl)-5-chloro-1-naphthalenesulfonamide (W7, 50 μM), a typical calmodulin antagonist, on CSQ1-STIM1 interaction. We found that unlike TFP, W7 did not further enhance the CSQ1-STIM1 association upon TG stimulation (Supplemental Fig. 3) and also did not raise the resting [Ca^2+^]_i_ in HEK293 cells^26^. Thus, these results indicate that TFP, by virtue of its ability to increase monomeric CSQ1, can enhance the interaction between CSQ1 and STIM1, prevent STIM1 redistribution on membrane, and thereby inhibit STIM1-Orai1 association and SOCE activation by TG.

### Asp-rich C-terminus of CSQ1 is responsible for CSQ1-STIM1 association

The CSQ carboxyl-terminus contains short runs of alternating negatively charged glutamate and aspartate residues in very high content^20^,^23^. Previous studies have shown that the asp-rich region of CSQ has dual functions of binding to Ca^2+^ and to triadin^18^,^19^, suggesting a general role in interactions with other proteins. To elucidate the STIM1 binding domain in CSQ1, we constructed plasmids encoding HA-tagged human wild-type *CSQ1* and two carboxyl-terminal deletion mutants: HA-tagged *CSQ1* lacking residues 397–405 (wild-type residues 388–396), referred as C9, and HA-tagged CSQ1 lacking residues 371–405 (wild-type residues 362–396), referred to as C35 (Supplemental Fig. 4A). Thirty-six hours after transfecting HEK293 cells with these mutants, the expression of the three exogenous HA-tagged CSQ1 was assessed by Western blot, and no significant difference in their expression levels was found (Supplemental Fig. 4B). Additionally, the relative abundances of CSQ1, STIM1 and Orai1 were also examined, and none of them were altered, except for an approximate 2.5-fold increase in total CSQ1 content for all the three exogenous CSQ1 expression vectors (Supplemental Fig. 4C).

To determine if and how the carboxyl-terminal amino acid deletions might alter CSQ1-STIM1 interaction, co-immunoprecipitation experiments were conducted to assess the association of HA-tagged CSQ1 with STIM1. In all three groups of transfected cells, the amount of STIM1 that co-immunoprecipitated with HA-tagged CSQ1 was determined following pull down with a specific anti-HA antibody (Fig. 2E). The strongest association was found in C9-expressing cells, followed by HA-CSQ1, with very little co-immunoprecipitation with the C35 cells upon store depletion. Similar results were obtained in the reverse co-immunoprecipitation experiment wherein HA-tagged CSQ1 association was evaluated following pull-down of STIM1 with a specific STIM1 antibody (Fig. 2F). These results suggest that the ablation of 35 carboxyl-terminal residues weakens CSQ1-STIM1 interaction, while the deletion of 9 carboxyl-terminal asp-rich residues unexpectedly intensifies their association compared with the full length CSQ1. In addition, these alterations in the CSQ1-STIM1 interaction led to an attenuation of the STIM1-Orai1 interaction in HA-CSQ1 and C9 constructed cells, while no significant effect was found with the C35 truncation (Fig. 2G). Thus, while all three constructs increased the total expression of CSQ1, only the constructs containing residues 362–396 interacted significantly with STIM1 and inhibited STIM1-Orai1 association.

**Figure 3:**
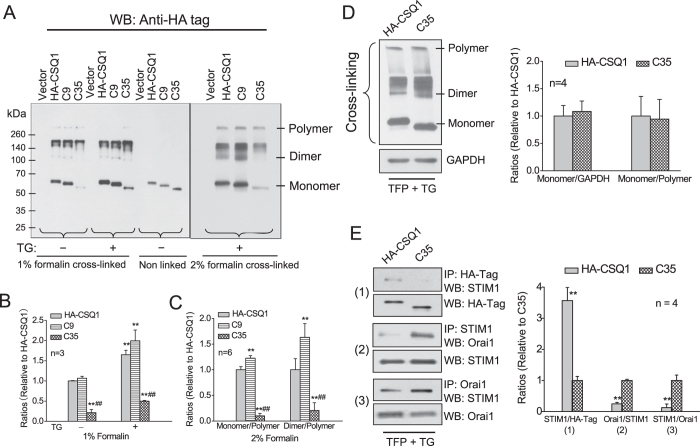
Conformation of CSQ1 in C terminal truncated cells. (**A**) Effects of carboxyl-terminal truncations on store depletion-induced CSQ1 conformation changes. HEK293 cells expressing various CSQ1 constructs were stimulated with or without 1 μM TG in Ca^2+^-free medium for 5 min and cross-linked with 1% or 2% formaldehyde for 5 min at room temperature. The cell lysates were separated by 10% SDS-PAGE and subjected to Western blot with anti-HA rabbit mAb. (**B** and **C**) The antibody-reacting bands of exogenous CSQ1 were quantified and the average ratios of monomer/polymer CSQ1 between control and TG-treated groups (**B**) and among all the three TG-treated groups (C) are represented as means ± SD. N = 3 and 6 for each bar in **B** and **C**. **p < 0.01 vs. HA-CSQ1; ^##^p < 0.01 vs. C9-CSQ1. (D) The combination of TFP and TG results in similar monomer formation in HA-CSQ1 and C35 cells. HEK293 cells expressing HA-CSQ1 or C35 were pre-incubated with 20 μM TFP for 10 min, changed to Ca^2+^-free medium for 5 min and then treated with 1 μM TG for another 5 min. Cells were cross-linked with 2% formalin for 5 min, and the cell lysates were separated by 10% SDS-PAGE and subjected to Western blot analysis (left). The average ratios of monomer/GAPDH and monomer/polymer are represented as means ± SD (right). N = 4 for each bar. (**E**) Similar CSQ1 monomers in HA-CSQ1 and C35-CSQ1 manifest different effects on CSQ1-STIM1 and STIM1-Orai1 associations. The whole cell lysates from experiments in (**D**) were immunoprecipitated with anti-HA, anti-STIM1 or anti-Orai1 antibody, and protein-A agarose followed by Western blotting analysis with anti-STIM1, anti-Orai1 or anti-STIM1 mAb, respectively. STIM1-exogenous CSQ1 association intensities were quantified as average protein ratios ± SD of STIM1/HA-Tag (1), and STIM1-Orai1 association intensities were quantified as average protein ratios ± SD of Orai1/STIM1 (2) and STIM1/Orai1 (3). N = 4, and **p < 0.01 vs. HA-CSQ1 for each bar.

Next, we sought to examine possible changes in CSQ1 structure conformation of the CSQ1 mutants. As shown in Fig. 3A, after cross-linking by 1% or 2% formaldehyde, polymeric and low-polymeric CSQ1 forms were detected in whole cell lysates. The exclusive monomer bands in the absence of cross-linking verified the effectiveness of formaldehyde cross-linking as well as the similar total expression of the three constructs. TG stimulation caused an increase in monomers accompanied by a reduction in polymer forms, and clearer polymer bands appeared upon increasing the formaldehyde concentrations from 1% to 2%. Among the three groups of transfected cells, the proportion of monomeric CSQ1 was the highest in C9-treated cells, followed by HA-CSQ1 and then C35 cells (Fig. 3B,C), consistent with the idea that CSQ1 associates with STIM1 as monomers. We also attempted to determine the transformation of CSQ1 upon TG stimulation in non-denatured lysates and gels, because of the possibility that the cross-linking procedure might have resulted in unwanted interactions with neighboring proteins, contributing to the apparent polymerization. However, in the native gels aggregation of the CSQ1 forms was actually substantially increased compared to that in the cross-linking experiments (Supplemental Fig. 4D), such that quantitation of the oligomeric forms was not possible.

The decrease in C35-CSQ1 monomers also raises the possibility that C35 gives less CSQ1-STIM1 interaction simply due to the lower proportion of CSQ1 monomers (Fig. 2). To further investigate this possibility, we used 20 μM TFP to induce CSQ1 monomerization, such that similar monomerization of CSQ1 was induced in both HA-CSQ1 and C35-CSQ1-treated cells (Fig. 3D). However, C35-CSQ1 still caused reduced CSQ1-STIM1 association and enhanced STIM1-Orai1 association under this condition (Fig. 3E), demonstrating that the distinct effects of C35 do not result entirely from the lowered CSQ1 monomers.

### CSQ1 inhibition of STIM1-Orai1 interaction inhibits SOCE

**Figure 4:**
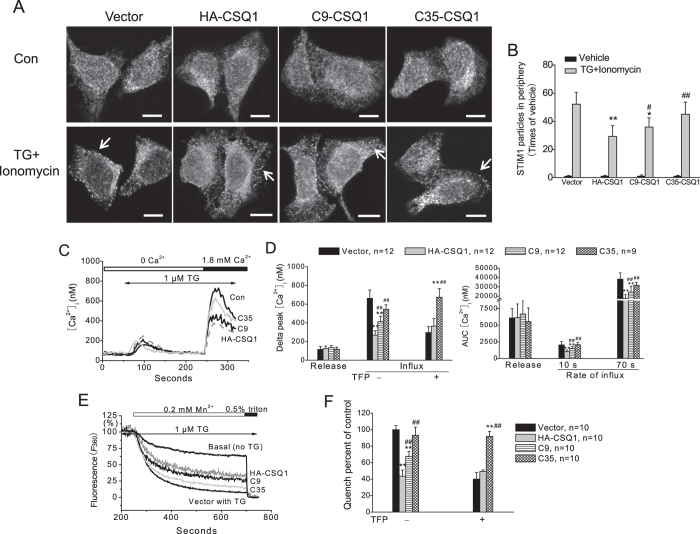
Exogenous expressions of HA-CSQ1 and its truncation mutants affect STIM1 redistribution and SOCE differently in HEK293 cells. (**A**) STIM1 aggregation and redistribution due to Ca^2+^ store depletion in HEK293 cells transfected with different plasmids. Scale bar = 10 μm. (**B**) The average density of STIM1 particles in the cell periphery induced by TG plus ionomycin (relative to vehicle control) are represented as means ± SD. N = 24–32 cells for each bar. (**C**) Effects of exogenous HA-CSQ1 or its truncation mutants on SOCE, representative traces. (**D**) Average peaks ± SD of release and influx from experiments shown in (**C**) and experiments not shown in which cells were preincubated with 20 μM TFP (left) and total amount of released Ca^2+^ (as area under the curve, AUC), and Ca^2+^ influx rate during the initial 10 s and 70 s in response to TG as indicated (right). N = 9–12 independent experiments for each bar. (**E**) Effects of exogenous HA-CSQ1 or its truncation mutants on Mn^2+^ influx in HEK293 cells. (**F**) Average Mn^2+^ quench rates for each condition ± SD from 10 independent experiments. *p < 0.05 and **p < 0.01 vs. vector group, and ^#^p < 0.05 and ^##^p < 0.01 vs. HA-CSQ1 group in panels **B**, **D** and **F**.

To investigate whether this regulatory mechanism plays a functional role in SOCE, STIM1 aggregation and Ca^2+^ influx induced by Ca^2+^ store depletion with TG were examined by immunostaining and Fura2 fluorescence. As shown in Fig. 4A,B, clustering and redistribution of STIM1 in cell periphery could be easily found in vector and C35-CSQ1 cells, whereas more aggregation in the cytosol and incomplete clustering in the cell periphery of STIM1 were found in HA-CSQ1 and C9-CSQ1 treated cells compared with that of vector group. Consistently, there was no significant difference in the Ca^2+^ release fraction among HA-CSQ1-, C9-CSQ1- and C35-CSQ1-treated cells. SOCE evaluated by peak and rate of Ca^2+^ entry (minus basal) was reduced by over-expression of HA-CSQ1 or C9-CSQ1 compared with vector control, but not by the C35-CSQ1 mutant (Fig. 4C,D). Additionally, in agreement with the observation found in STIM1-Orai1 interaction (Fig. 3E), TFP (20 μM) could not restore the inhibitory effect of C35-CSQ1 (Fig. 4D), despite a similar level of monomeric CSQ1 in C35-CSQ1 cells compared to HA-CSQ1 cells (Fig. 3D). In addition, SOCE in these transfected cells was assessed by the Mn^2+^ quenching assay to more clearly examine the divalent cation influx uninfluenced by Ca^2+^ buffers and transport mechanisms. A basal quench was observed upon 0.2 mM Mn^2+^ addition in Ca^2+^-free medium. After TG (1 μM) depletion of ER Ca^2+^ stores, addition of Mn^2+^ resulted in a more rapid fluorescence decrease, due to SOCE^30^,^31^. After normalization to the basal Mn^2+^ quench, the fluorescence decrease induced by TG in HA-CSQ1 group appeared to be the smallest followed by C9 and then C35 groups when compared with control group (Fig. 4E,F), consistent with the results from Ca^2+^ influx experiments. As seen in the Ca^2+^ influx experiments, TFP failed to restore the inhibitory effect of C35-CSQ1 on Mn^2+^ influx (Fig. 4F).

### CSQ1 knockdown enhances Ca^2+^ responses to TG

Finally, the Ca^2+^ changes in cells with reduced endogenous CSQ1 expression by siRNA plasmid transfection (Supplemental Fig. 5A) were determined. This resulted in enhanced resting [Ca^2+^]_i_, Ca^2+^ release and Ca^2+^ influx responses to TG (0.5 μM) compared with those in control siRNA-treated cells (Supplemental Fig. 5B,C). Furthermore, consistent with previous findings^26^, the increased basal [Ca^2+^]_i_ was found to be sensitive to 2-APB (30 μM) addition (data not shown), a compound commonly used to inhibit SOCE.

## Discussion

Previous studies have shown that CSQ1 regulates SOCE, and that the aspartate-rich segment is responsible for its interference with the SOCE pathway in skeletal myocytes^24^,^25^. Our results demonstrate that CSQ1 physically interacts with STIM1. Upon TG stimulation, CSQ1-STIM1 and STIM1-Orai1 associations are increased synchronously (Fig. 1A,B), implying that CSQ1 is likely to be involved in the regulation of the interaction between STIM1 and Orai1. These results are further confirmed by TFP treatment, which has been found to modulate CSQ conformation, inhibiting Ca^2^-induced folding and aggregation of CSQ molecules^21^,^28^. Here, through the enhancement of CSQ1 monomerization (Fig. 1C,D), TFP promoted CSQ1 association with STIM1 (Fig. 2A), but weakened the STIM1-Orai1 interaction (Fig. 2B) and SOCE in TG-treated cells (Fig. 2C,D), which further supports the conclusion that CSQ1 restrains SOCE via association with STIM1. In addition, we show that TG also elevated the proportion of monomeric CSQ1 and the CSQ1-STIM1 association that accompanies the STIM1-Orai1 interaction (Fig. 1A–D). This implies that store depletion not only activates STIM1-Orai1 interaction, but also STIM1-CSQ1 interaction which in turn acts as a negative regulator of SOCE (Fig. 2). This conclusion is supported by the observation that a decrease in endogenous CSQ1 expression promotes SOCE in HEK293 cells (Supplementary Figure 5) and skeletal muscle cells^25^.

The aspartate-rich carboxyl-terminus of CSQ1 has been reported to have multiple functions to bind Ca^2+^ as well as triadin/junctin and to act as a suppressor of SOCE^17^,^18^,^19^,^20^,^21^,^25^. Our data utilizing truncation mutants of CSQ1 demonstrate that the carboxyl-terminal 35 residues are necessary for CSQ1-STIM1 association (Figs 2 and 3). Additionally, this sequence is also located within the domain responsible for CSQ1 monomerization, because a dramatic blockade and an augmentation of monomerization are found in C35-CSQ1- and C9-treated cells, respectively (Fig. 3A–C). The latter effect may result from substantially reduced steric hindrance for protein-protein association^32^, or alternatively, from reduced Ca^2+^ binding causing more monomer CSQ1 formation^18^,^19^,^20^,^21^, and thus an enhancement of CSQ1-STIM1 interaction (Fig. 2E–G). According to previous studies, large surface areas are buried in the formation of polymers through intricate interactions^33^,^34^. Therefore CSQ1 conformation changes can provide the availability of domains responsible for protein-protein binding, in which monomeric forms may present a greater number of exposed binding sites for STIM1, and thereby attenuate STIM1 interaction with Orai1 (Figs 2 and 3). Interestingly, this inhibitory effect of monomer CSQ1 disappears in cells with deletion of the carboxyl-terminal 35 residues of CSQ1 even in the presence of TFP, but not with deletion of the last 9 residues (Figs 2 and 3). This suggests that the binding site for STIM1 resides within the CSQ1 carboxyl-terminal residues 362–387 (Fig. 2E–G) that are also important for CSQ1 monomerization (Fig. 3A–C). Residues 388–396 are important for CSQ1 compaction (Fig. 3A–C), but also lie within the motif of interaction with STIM1 since the enhanced C9-STIM1 association does not lead to diminished STIM1-Orai1 interaction and SOCE as might be expected when compared to wild-type CSQ1 (Figs 2, 3, 4). Hence, we speculate that deletion of C9 enhances monomer formation thus increasing STIM1 association, but also weakens the STIM1-CSQ1 interaction resulting in minimal change in STIM1-Orai1 interaction. Truncation of C35 weakens both binding and interacting with STIM1, providing a more pronounced inhibitory effect on STIM1 oligomerization and the ensuing STIM1-Orai1 association.

**Figure 5:**
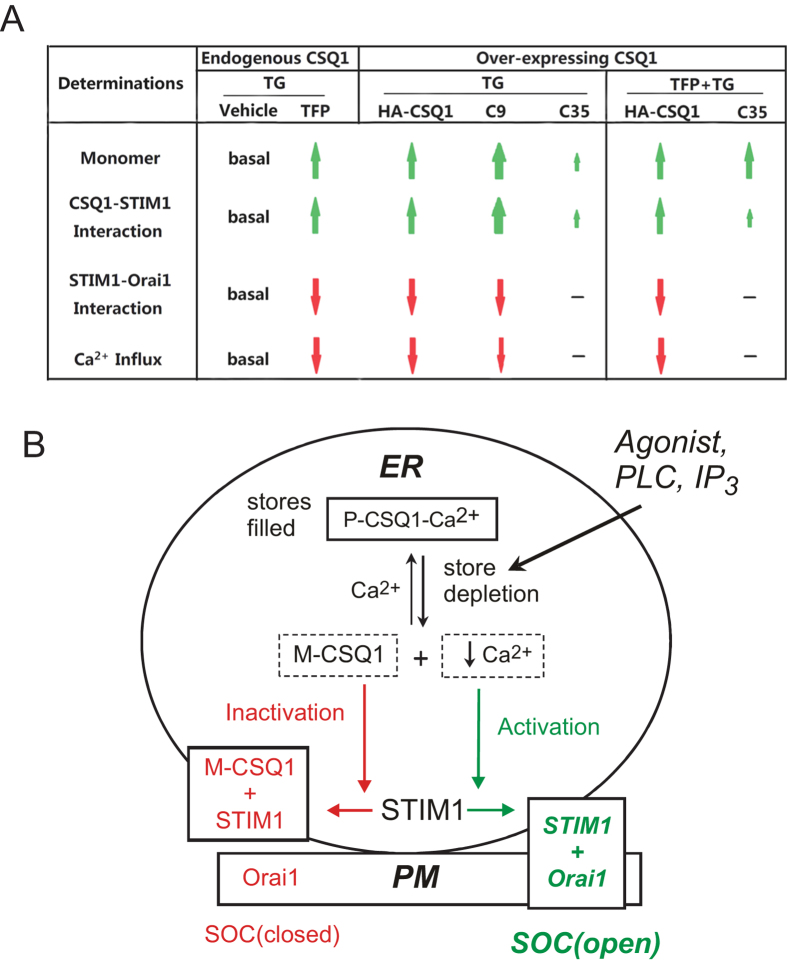
Proposed model for regulation of SOCE by CSQ1. (**A**) The effects of TFP, HA-CSQ1 and the truncation mutants on CSQ1 monomerization, interactions of CSQ1-STIM1 and STIM1-Orai1 and TG-induced Ca^2+^ influx are summarized. (**B**) A schematic depicts the negative regulatory role of CSQ1 in SOCE signaling. In the resting state, stores are filled with Ca^2+^ that binds to polymeric CSQ1 (P-CSQ1). Upon store depletion, the lowered Ca^2+^ in ER activates two simultaneous pathways in the ER: 1) STIM1 oligomerizes and interacts with Orai1 to activate SOCE, and 2) CSQ1 monomerizes (M-CSQ1) and interacts with STIM1, thus reducing STIM1 binding to Orai1 and braking SOCE. See text for more details. PLC, IP_3_, ER and PM stand for phospholipase **C**, inositol 1,4,5-trisphosphate, endoplasmic reticulum and plasma membrane.

On the basis of these results we propose a model for the regulation of SOCE by CSQ1 (Fig. 5). ER Ca^2+^ stores are full in the resting state, and CSQ1 linear polymers binding large amounts of Ca^2+^ are anchored in the vicinity of Ca^2+^ release channels^21^,^34^, likely the IP_3_ receptor channels in the case of non-excitable cells. There is a dynamic equilibrium of dissociation and compaction between CSQ1 polymers and monomers, in which polymers should predominate due to the abundant ER Ca^2+^. In this condition, a small fraction of monomers bind to undefined luminal sites on STIM1. Upon ER store depletion, CSQ1 polymers dissociate into low-polymeric forms, such as dimers and monomers, which in turn increases binding of CSQ1 to STIM1. Meanwhile, depletion of Ca^2+^ also results in Ca^2+^ dissociation from the STIM1 EF-hand motif, which causes STIM1 oligomerization, and then migration, aggregation and accumulation in pre-existing ER-PM junctions. Finally, the direct binding of STIM1 to Orai1 recruits Orai1 into puncta resulting in localized Ca^2+^ influx. However, this Ca^2+^ influx is somewhat limited by CSQ1 sequestration of STIM1 that may somehow inhibit clustered STIM1 movement to the cell periphery (Fig. 4A,B). Thus, CSQ1 is similar to STIM1 in that both of them undergo conformational changes as a result of Ca^2+^ store depletion, and both of the ER proteins are involved in SOCE pathway signaling. However, STIM1 is as an activator of store-operated Orai1 channels, while CSQ1 appears to function as a down-regulator for SOCE, preventing excessive Ca^2+^ entry upon stimulation^25^ and also at resting state^26^,^28^ (Supplementary Figure 5). A recent finding that a complex of JP45, a membrane protein, and CSQ1 modulates Cav1.1 channel activity in muscle fibers^35^ suggests that this modulatory function of CSQ1 on plasma membrane Ca^2+^ channel activity may represent a more general cellular function of CSQ1.

Signal transduction occurring through CSQ1 dissociation in the narrow ER space is likely to be of considerable significance because under physiological conditions, endogenous stimulators such as neural transmitters and hormones produce Ca^2+^ mobilization and activate SOCE as a significant component of their signaling repertoire. In this context, CSQ1 conformational changes presumably occur (Fig. 1D)^19^,^20^,^22^, thereby acting as a physiological regulator of SOCE. Additionally, a number of pathological conditions characterized by overload of Ca^2+^ and abnormal Ca^2+^ activity in cells are known to result from deletion or loss-of-function in calsequestrins; these include malignant hyperthermia and catecholaminergic polymorphic ventricular tachycardia^22^,^23^,^36^,^37^. Although these conditions are commonly linked to the loss of Ca^2+^ buffering by CSQ, loss of regulation of SOCE will also surely occur.

In summary, here we report, for the first time, a functional interaction between CSQ1 and STIM1 that occurs simultaneously with SOC activation by Ca^2+^ store depletion. This association follows monomerization of CSQ1 due to lowered ER Ca^2+^, and inhibits STIM1 interaction with Orai1 and thus SOCE activation. In addition, we show that the site of binding and interaction of CSQ1 with STIM1 lies within, or overlaps with the carboxyl-terminal 362–387 and 362–396 residues of CSQ1, respectively. Thus, this study demonstrates a novel signaling pathway providing Ca^2+^ store-dependent negative regulation of STIM1-Orai1-mediated Ca^2+^ entry and reveals a new level of complexity in the SOCE pathway.

## Methods

### Reagents and antibodies

Fura2-AM, Lipofectamine 2000, Protein A agarose, rProtein G agarose, Alexa fluor dye-conjugated secondary antibodies were obtained from Life Tecnologies. Super Signal West Pico enhanced chemiluminescence reagents kit was from Thermo Scientific (MA, USA). Formaldehydrate solution, thapsigargin, trifluoroperazine dihydrochloride, (N-(6-aminohexyl)-5-chloro-1-naphthalenesulfonamide hydrochloride, protease inhibitor cocktail were from Sigma-Aldrich (St. Louis, MO, USA). HA-tag mouse monoclonal antibody (Ab24779) was from Abcam(Cambridge, USA). All of the other primary and secondary antibodies including mouse monoclonal anti-CSQ1 (SC-137080), goat polyclonal anti-CSQ2 (SC-16576), rabbit polyclonal anti-CSQ1 (SC-2005), mouse monoclonal anti-STIM1 (SC-166840), mouse monoclonal anti-Orai1 (SC-377281), rabbit polyclonal anti-HA tag (SC-805), mouse monoclonal anti-actin (SC-2005), mouse monoclonal anti-GAPDH (SC-2005), goat anti-mouse IgG-HRP (SC-2005), goat anti-rabbit IgG-HRP (SC-2004) were from Santa Cruz Biotechnology, Inc. (Santa Cruz, CA, USA).

### Cell culture

HEK293 cells were purchased from ATTC and cultured in DMEM (Gibco, Rockville, MD, USA) supplemented with 10% fetal bovine serum (FBS, Hyclone, AUS) at 37 °C under 95% air and 5% CO_2_. Plasmids were transiently transfected into HEK293 cells by Lipofectamine 2000 reagent following the manufacturer’s protocol using 2 μg/ml in DMEM of each construct.

### Plasmids and cell transfections

Plasmids were constructed by Life Technologies CO.LTD (AB & Invitrogen). HA-tagged human wild-type CSQ1 and its mutant cDNA were generated by PCR. Each cDNA were cloned into the pIRES2-EGFP expression vector. HA tag (YPYDVPDYA) was inserted downstream of the predicted signal sequence of the wild-type *CSQ1* between Val49 and IIe50. The deletion mutants were C9 (deletion of wild-type amino acids 388–396) and C35 (deletion of wild-type amino acids 362–396). Nucleotide sequences of these constructs were verified by sequencing. These constructs (20 μg each) were transfected into HEK293 cells for 36 h and protein expression was confirmed by Western blot analysis. Briefly, cells were seeded 24 h ahead into 10 cm dishes with antibiotics-free, 10% FBS-containing DMEM and allowed growing to 80% confluence. We diluted 20 μg plasmid DNA and 20 μl Lipofectamine 2000 reagent with FBS-free DMEM to 500 μl in two separated tubes and incubated for 5 min at room temperature, and then added the diluted DNA to diluted Lipofectamine 2000 reagent and mixed by gently inverting the tubes 3–5 times and incubated the DNA-lipid complex for 15 min at room temperature. We replaced the growth medium with 9 ml FBS-free DMEM in the culture dish and added 1 ml DNA-lipid complex to cell dishes. After 4 h incubation at 37 °C under 95% air and 5% CO_2_, we changed them with 10% FBS-containing DMEM and cultured the cells for 36 h before harvest. Through the whole process, the DMEM medium was antibiotic-free.

CSQ1 knockdown siRNA (CSQ1-KD) and non-specific control siRNA (Con-siRNA) plasmids were constructed by Life Technologies CO.LTD (AB & Invitrogen). They were transfected into HEK293 cells as previously reported^26^.

### Western Blotting

Cell protein extracts were prepared by washing the cells with PBS and then lysing the cells with lysis buffer, supplemented with freshly added 1 mM PMSF, protease inhibitor cocktail. Protein samples were denaturized by incubation in loading buffer at 95 °C for 5 min. Proteins were resolved on 10% SDS-PAGE and electroblotted onto nitrocellulose membranes. The membranes were subsequently blocked for 1 h at room temperature in 5% non-fat milk with Tris-buffered saline/Tween-20 (TBST), pH 7.4. Then the membranes were incubated overnight at 4 °C with anti-CSQ1 (1:1000), anti-STIM1 (1:1000), anti-Orai1 (1:1000), anti-HA (1:5000) primary antibodies for detection of CSQ1, STIM1, Orai1, HA-tag, respectively. After washing with TBST, membranes were incubated with horseradish peroxidase-conjugated secondary antibodies (1:2000) at room temperature for 1 h and then exposed to Super Signal West Pico enhanced chemiluminescence reagents for 2 min. Blots were then exposed to Kodak photographic films. The gray scale intensity of bands was measured using densitometry software Quantity One from Bio-Rad.

Non-denaturing were treated in the sample buffer containing 150 mM sodium chloride, 0.2% Triton X-100 and 50 mM Tris, pH 8.0. All the samples were then subjected to non-denaturing electrophoresis using a resolving gel containing 10% (w/v) acrylamide, 0.33% bisacrylamide, 10% ammonium persulphate, 0.1% N,N,N’, N’-tetramethylethylenediamine (TEMED), 1.5 M Tris, pH 8.8. The stacking gel contained 5% (w/v) acrylamide, 0.15% bisacrylamide, 0.07% ammonium persulphate, 0.1%TEMED, and 1 M Tris, pH 6.8. The electrode buffer contained 25 mM Tris/glycine buffer, pH 8.5. The gel was run at 4 °C for 4 h at 80 V. Gels were elctrolotted on nitrocellulose membranes. Then the membrane was treated as described above.

### Co-immunoprecipitation

Cells were harvested, washed with PBS and then lysed with lysis buffer: supplemented with freshly added 1 mM PMSF, protease inhibitor cocktail. Then the lysates were centrifuged at 4 °C, 15000 g for 15 min. The supernatants were precleared with rProtein G (for mouse monoclonal primary antibodies) or Protein A (for rabbit polyclonal primary antibodies) beads and protein concentration was determined, using the Pierce BCA Protein Reagent Kit. The cell extracts were then adjusted to approximately 1–2 mg/ml total protein with lysis buffer and mixed with corresponding primary antibodies (0.2 μg/100 μg proteins) as indicated and incubated at 4 °C overnight under constant agitation. Equal amounts of samples were mixed with normal IgG as negative controls. Beads were subsequently added to the mixtures (≥5 μl per 1 μg IgG) and agitation was continued for 4 h at 4 °C. The beads were then washed with lysis buffer and proteins were eluted by incubation in loading buffer at 95 °C for 5 min. The eluted proteins were resolved on 10% SDS-PAGE and analyzed by Western blotting as described above.

### Immunofluorescence

Cells were plated on 10 × 10 mm cover slides in 6 well plates (5 × 10^4^ cells per well). After 24 h, various treatments were applied to the cells, and they were then fixed in phosphate-buffered saline (PBS) containing 4% paraformaldehyde for 15 min. The cells were then permeabilized by 0.5% Triton X-100 in PBS for 30 min and blocked in 5% bovine serum albumin for 30 min at room temperature. The primary antibodies were diluted (1:400) in PBS and added to the permeabilized cells, which were incubated overnight at 4 °C. Dye-conjugated secondary antibodies (1:400, Alexa Fluor, Invitrogen) were added for 1 h at room temperature. For nuclear staining, cells were incubated with Hochest33342 for 10 min at room temperature. After mounting in mounting medium, a 3D Z-stack was acquired at 0.5 μm intervals by Leica SP8 microscopy equipped with a 63× oil immersion objective (NA 1.4), pinhole = 1 μm. For the measurement of STIM1 aggregation, HEK293 cells expressing empty vectors or exogenous HA-CSQ1, C9 or C35 truncations were suspended in Ca^2+^-free HBSS and then stimulated with 1 μM TG plus 5 μM ionomycin for 5 min. Then the cells were immuno-labeled with mouse anti-STIM1  mAb at a dilution of 1:400. Alexa Fluor 594 goat-anti-mouse secondary antibody was used at 1:400. The bottom of each cell was firstly determined, and then the x-y image of the plane 3 μm above the bottom was taken by Leica SP8 microscopy, the pinhole = 1.5 μm. The selection of STIM1 particles in the cell periphery was performed by Leica Qwin Standard V2.8, and the number in each cell was normalized to the cell circumference, and then compared between groups.

### Chemical Cross-linking

As previously described^38^,^39^, HEK293 cells were collected by centrifugation at 300 g for 1 min and resuspended in Ca^2+^-free PBS. Cells were then cross-linked with 1% or 2% (w/v, as indicated) formaldehyde solution for 5 min. The cross-linking reaction was terminated by the addition of a 1 M glycine stock solution, pH 8.0, to a final concentration of 0.1 M and incubation was continued for 5 min on ice. Cells were collected by centrifugation at 300 g for 1 min and washed 3 times with ice-cold PBS. Then cells were lysed with lysis buffer supplemented with 1% SDS, 0.1% Triton-X100, 1 mM PMSF, 10 μl protease inhibitor cocktail under constant agitation for 5 min at 4 °C. The lysates were then centrifuged at 4 °C, 15000 g for 15 min and the supernatants were collected for protein concentration assay and Western blotting without boiling before loading in the comb hole.

### Measurement of [Ca^2+^]_i_

Cytosolic free Ca^2+^([Ca^2+^]_i_) was measured using the fluorescent probe Fura2. HEK293 cells suspensions were loaded with Fura2 by incubation in culture medium supplemented with 1 μM Fura2 acetoxy methyl ester at 37 °C for 30 min. After washing by centrifugation with 1.8 mM Ca^2+^-containing Hepes-buffered saline solution (HBSS) and then Ca^2+^-free HBSS, cells were resuspended in Ca^2+^ free HBSS for subsequent use in experiments. The fluorescence was monitored in a stirred cuvette with fluorescence spectrophotometer HITACHI F-7000 at 37 °C. The excitation wavelengths were alternated between 340 and 380 nm and emission was measured at 510 nm. At the end of each incubation, a final concentration of 0.2% Triton X-100 and 5 mM EGTA were added to obtain maximal (*F*_max_) and minimal (*F*_min_) fluorescence, respectively. Values of [Ca^2+^]_i_ were calculated from the ratios of fluorescence with excitation at 340 nM to that at 380 nM by the FL Solutions Intracellular Cation Scan software based on the formula described by Grynkiewicz^40^. The quenching of Fura2 fluorescence by Mn^2+^ (0.2 mM) in 1 μM TG-treated cells expressing HA-CSQ1 or either of the two truncation mutants was measured at the Ca^2+^-independent excitation wavelength of Fura2 (360 nm) in Ca^2+^-free HBSS. Cells were treated with 1 μM TG for 250 s to deplete ER Ca^2+^ stores, and were pre-incubated with 20 μM TFP for 10 min or not as indicated. The initial rate (50 s) of decline of Fura2 fluorescence upon Mn^2+^ addition was calculated and expressed as percent of the rate for the vector control (-TFP). The maximally quenched fluorescence signal was achieved at the end of the experiment by lysing the cells with 0.5% Triton X-100 (as 0%). Basal rate (no TG) was subtracting before calculating each rate, and the vector rate was then set as 100%.

### Mn^2+^ quenching

The rate of divalent cation influx unaffected by Ca^2+^ pumps was assessed by monitoring the rate of quenching of intracellular Fura2 by Mn^2+^ entering the cells. Transfected HEK293 cells were loaded with 2 μM Fura2-AM in DMEM for 30 min at 37 ^o^C and then washed in 1.8 mM Ca^2+^-containing HBSS and then Ca^2+^-free HBSS as for [Ca^2+^]_i_ measurements. The fluorescence was monitored in a stirred cuvette with fluorescence spectrophotometer HITACHI F-7000 at 37 °C. The excitation wavelength was set at the isosbestic point 360 nm and emission was measured at 510 nm. Following a 60 s stabilization period, 1 μM TG was added for 250 s, followed by 0.2 mM Mn^2+^ for 450 s. Subsequent addition of 0.5% Triton X-100 allows complete quench of Fura2 to obtain a background value.

### Statistical analysis

Data are presented as the means ± SD of three to twelve independent measurements. Statistical comparisons between groups were carried out with a two-tailed unpaired Student’s t-test followed by one-way analysis of variance (ANOVA) as appropriate. P < 0.05 was considered statistically significant.

## Additional Information

**How to cite this article**: Wang, L. *et al.* Retrograde regulation of STIM1-Orai1 interaction and store-operated Ca^2+^ entry by calsequestrin. *Sci. Rep.* **5**, 11349; doi: 10.1038/srep11349 (2015).

## Supplementary Material

Supplementary Information
