# Supplementary material for: Retrograde regulation of STIM1-Orai1 interaction and store-operated Ca2+ entry by calsequestrin
Source: Sci Rep. 2015 Jun 18;5:11349. doi: 10.1038/srep11349 (PMC4471903; doi:10.1038/srep11349)
Supplement: Supplementary Information [file srep11349-s1.pdf]

Supplementary Materials

Retrograde regulation of STIM1-Orai1 interaction and store-operated Ca^{2+} entry by calsequestrin

Limin Wang, Shu Li, Lane Zhang, Yuanyuan Zheng, Xinxin Yan, Min Chen, Haoyang Wang, James W. Putney, Dali Luo

Supplemental Figures Reference List

1. Costes,S.V., Daelemans,D., Cho,E.H., Dobbin,Z., Pavlakis,G., & Lockett,S. Automatic and quantitative measurement of protein-protein colocalization in live cells. *Biophys. J.* 86, 3993-4003 (2004).

2. Sugawara,Y., Kamioka,H., Honjo,T., Tezuka,K., & Takano-Yamamoto,T. Three-dimensional reconstruction of chick calvarial osteocytes and their cell processes using confocal microscopy. *Bone* 36, 877-883 (2005).

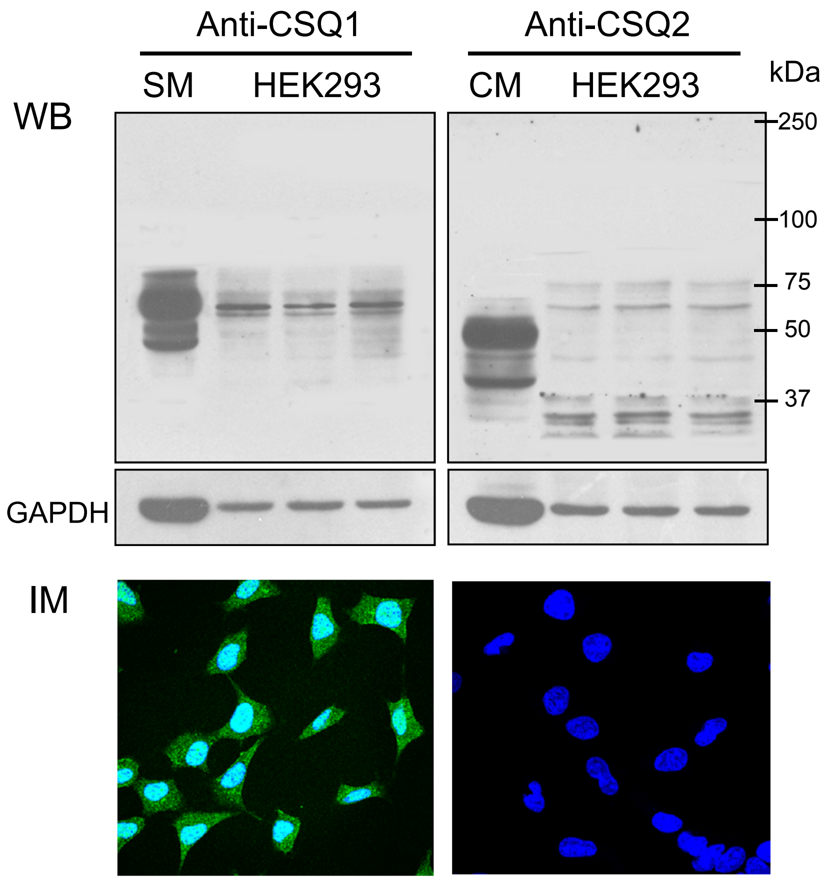

Supplemental Figure 1. HEK293 cells express CSQ1 but not CSQ2 subtype. HEK293 cells were cultured for 24 h and lysed with RIPA buffer. The whole cell lysates were immunoblotted (WB: upper images) and immunostained (IM: bottom images) with specific anti-CSQ1 and anti-CSQ2 antibodies, respectively. Cell nucleuses are indicated by Hoechest 33258 (1 µg/ml). SM and CM stands for skeletal muscle and cardiac muscle, respectively. Scale bar = 10 µm. Similar results were obtained in three independent experiments.

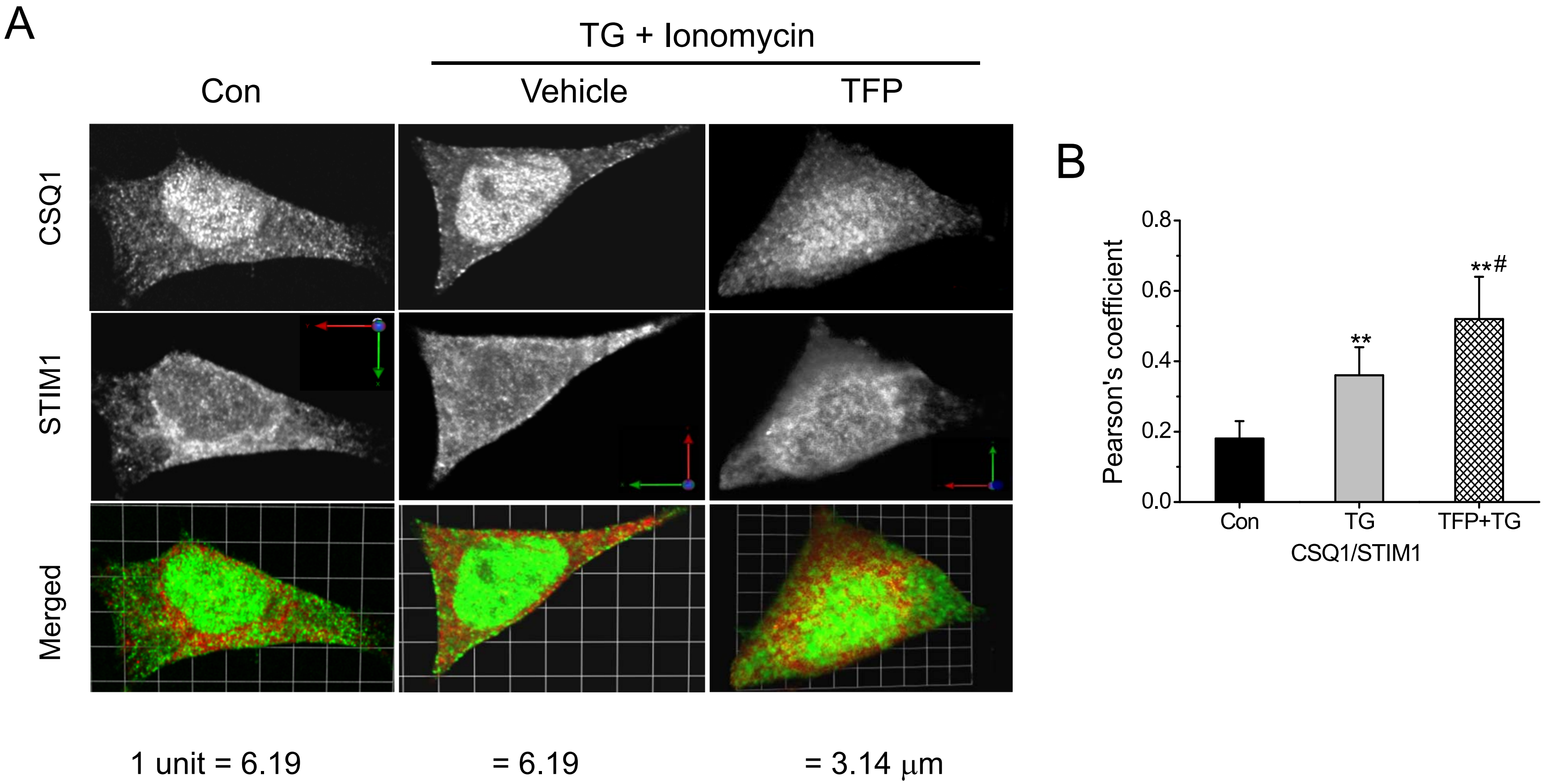

Supplemental Figure 2. Immunohistochemical co-localization of endogenous CSQ1 and STIM1. HEK293 cells cultured for 24 h were incubated in FBS-free DMEM with or without the presence of 20 μM TFP for 10 min, and then were incubated in Ca^{2+}-free HBSS for 5 min and stimulated with 1 μM TG plus 5 μM ionomycin for another 5 min. The control cells followed the same procedures but without any treatment. Then cells were immuno-labeled with mouse anti-STIM1mAb and rabbit polyclonal anti-CSQ1 antibody at a dilution of 1:400 for both as described in Methods. Alexa Fluor 594 goat-anti-mouse and Alexa Fluor 488 goat-anti-rabbit secondary antibody were used at 1:400. (A) Representative confocal bottom views from three-dimensional visualizations of HEK293 cells demonstrate colocalization of STIM1 and CSQ1 is increased by TG. This co-localization is further increased by TFP, while the near plasma membrane concentration disappears. (B) Analysis of co-localization by Pearson's coefficient in the whole cell volume excluding nucleus using Imaris software[1,2] exhibits a basal co-localization of CSQ1 and STIM1 in control cells. Both TG and TG+TFP treatments enhanced their co-localization. **$p<0.01$ vs. control (vehicle) cells, n = 25-42 cells in 3 independent experiments. **$p<0.01$ vs. control, and $^{\#}p<0.05$ vs. TG+ionomycin treatment.

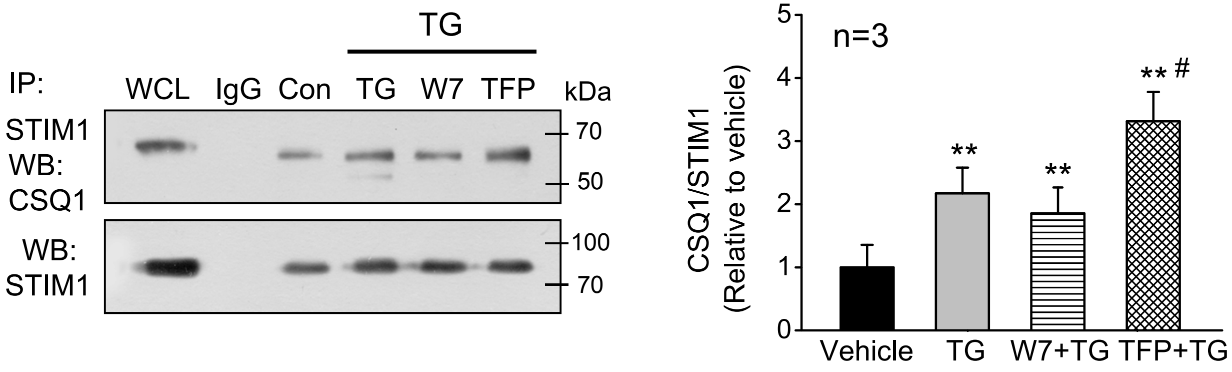

Supplemental Figure 3. Different effects of TFP and W7 on the association of CSQ1 and STIM1 in HEK293 cells. HEK293 cells were preincubated with or without 20 μM TFP or 50 μM W7 for 10 min, then were suspended in Ca^{2+}-free HBSS for 5 min and stimulated with 1 μM TG for another 5 min. Whole cell lysates (WCL) were immunoprecipitated with specific anti-STIM1mAb and protein-G agarose, then Western blot was performed with specific anti-CSQ1mAb. Equal amounts of normal mouse IgG was used as an immunoprecipitation antibody substitute for negative control, and membranes were reprobed with the antibody used for immunoprecipitation for protein loading control (left). CSQ1-STIM1 association intensities were quantified as average protein ratios ± SD of CSQ1/STIM1 (right). N = 3 for each bar; $^{**}p<0.01$ vs. Con (no any treatment), and $^{\#}p<0.05$ vs. TG group.

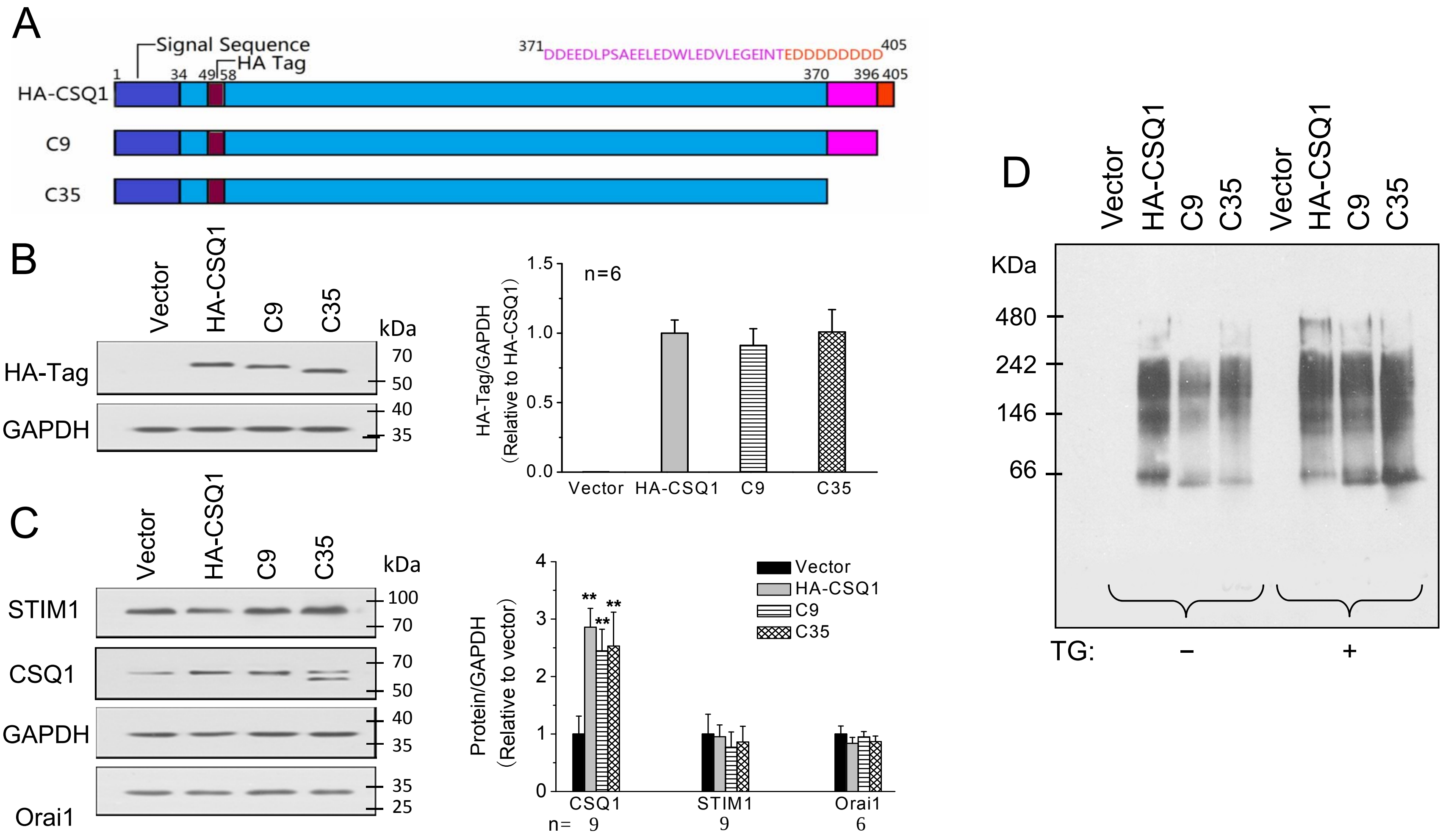

Supplemental Figure 4. Effects of carboxyl-terminal deletion mutants on SOCE associated protein expression. (A) Schematic of HA-Tagged wild-type human CSQ1 and CSQ1 truncations. HA-CSQ1 containing residues 1-405, C9: 1-396, C35: 1-370. (B) Exogenous CSQ1 expressions in HEK293 cells transfected with HA-CSQ1 and deletion mutants. The various CSQ1 plasmids were transfected into HEK293 cells for 36 h. Total cell lysates were separated by 10% SDS-PAGE and subjected to Western blot analysis with anti-HA rabbit mAb (left). The levels of exogenous CSQ1 expression were quantified as average ratios ± SD of CSQ1/GAPDH (right). (C) Expression of SOC-associated proteins and CSQ1 (endogenous and HA-Tagged) before and after transfection with exogenous HA-CSQ1 and deletion mutants. Representative Western blots for total CSQ1, STIM1, Orai1 and GAPDH in different plasmid treatment groups as indicated (left). The abundances of these proteins were quantified as average ratios ± SD of proteins/GAPDH (right). (D) Effects of carboxyl-terminal truncations on store depletion-induced CSQ1 conformation changes in non-denatured condition. HEK293 cells expressing various CSQ1 constructs were stimulated with or without 1 μM TG in Ca^{2+}-free medium for 5 min. The non-denatured cell lysates [see Methods] were separated by 10% SDS-PAGE and subjected to Western blot with anti-HA rabbit mAb.

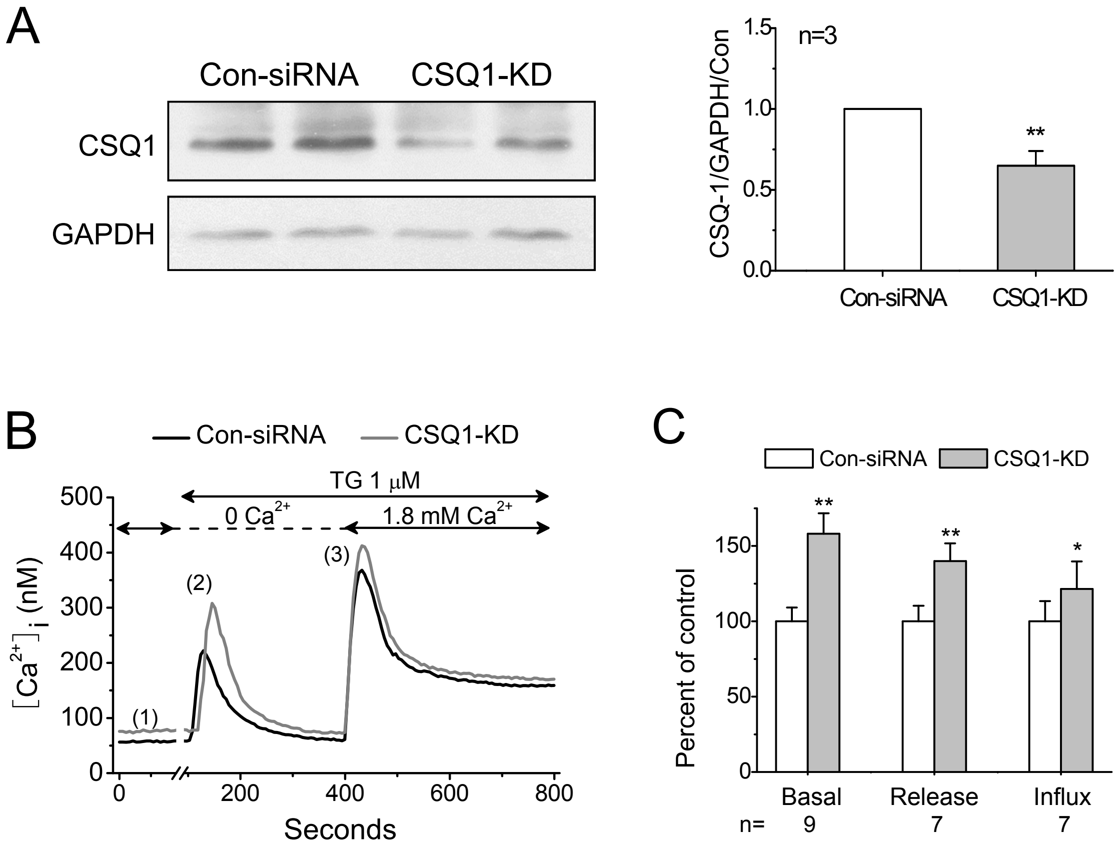

Supplemental Figure 5. Effects of CSQ1 knockdown on the basal internal Ca^{2+} and Ca^{2+} response to TG. (A) Endogenous expressions of CSQ1 in HEK293 cells transfected with empty control or CSQ1-KD siRNA. Plasmids carrying control or CSQ-KD gene were transfected into HEK293 cells for 42 h. Total cell lysates were separated by 10% SDS-PAGE and subjected to Western blot analysis with anti-CSQ1 rabbit mAb (left). The levels of endogenous CSQ1 expression were quantified as average ratios ± SD of CSQ1/GAPDH (right). (B) Effects of endogenous CSQ1 knockdown on SOCE. Representative traces depict the resting $[Ca^{2+}]_i$ level (1) in Ca^{2+}-containing medium and 0.5 μM TG-induced Ca^{2+} mobilization in Ca^{2+}-free HBSS (2) and Ca^{2+} influx monitored with a Ca^{2+} add-back protocol (3) in Fura2-loaded cells with deficient CSQ1. (C) Average peaks ± SD of resting Ca^{2+}, and Ca^{2+} release and influx to TG from experiments shown in (B). N = 7-9 independent experiments for each bar. *p<0.05 and **p<0.01 vs. Con-siRNA cells, n = 7-9 independent experiments.
